# OA05 A case of VEXAS syndrome treated with hematopoietic stem cell transplantation

**DOI:** 10.1093/rap/rkae117.005

**Published:** 2024-11-05

**Authors:** Helen Sugden, Sanah Sajawal, Sinisa Savic, Michael Green

**Affiliations:** York Teaching Hospitals Trust, York, United Kingdom; Leeds Teaching Hospitals Trust, Leeds, United Kingdom; National Institute for Health Research, Leeds, United Kingdom; York Teaching Hospitals Trust, York, United Kingdom

## Abstract

**Introduction:**

VEXAS is an acquired auto-inflammatory condition first described in 2020. It is linked to a somatic inactivating mutation in the X-linked UBA1 gene in haemopoietic progenitor cells. The UBA1 gene codes for UBA1 enzymes including E1 enzymes that catalyse the first steps of ubiquitination and lend an ‘E’ to the VEXAS acronym. Ubiquitination is a process whereby proteins are flagged for degradation, transported within the cell, or up or downregulated. We discuss the case of a 68-year-old male diagnosed with VEXAS syndrome and his response to treatments including glucocorticoids, biologic therapy, and stem cell transplantation.

**Case description:**

The patient presented with coryza, cough, night sweats, fever, and testicular pain. Bloods showed normocytic anaemia (Hb 128 g/L), neutropenia (neutrophils 1.9x10^9/L) and raised CRP (98 mg/L). PET CT, autoantibody testing, complement, ACE, CK, myeloma and infection screening were unremarkable. Symptoms progressed to include auricular and nasal swelling in keeping with chondritis, sinusitis, headaches, and myalgias. A diagnosis of relapsing polychondritis was suspected.

Treatment with prednisolone 40 mg once daily and methotrexate 15 mg once weekly commenced with dramatic biochemical (CRP 10 mg/L) and symptomatic improvement, though cytopenias persisted. Prednisolone was weaned gradually to 15 mg once daily, however, symptoms returned and remained uncontrolled despite reinstating higher steroid doses.

A bone marrow biopsy was undertaken and UBA1 mutation c.122T>c, p.Met41Th was found, in keeping with VEXAS syndrome. Treatment was escalated to tocilizumab with initial partial symptomatic response, however, cell counts continued to drop with Hb, platelets, and neutrophils dipping as low as 63 g/L, 23x10^9/L, and 0.4x10^9/L respectively. He required regular blood transfusions, developed severe and recurrent infections, and had rash, paraesthesias, scleritis, breathlessness, episodic tongue and throat swelling, and injection site reactions.

Following discussion about further treatment options, the patient went on to have a successful hematopoietic stem cell transplant (HSCT).

**Discussion:**

The inflammatory and haematological manifestations of VEXAS syndrome are frequently resistant to DMARD and biologic treatment which is reflected in this case. Corticosteroid use has been shown to improve inflammatory symptoms at high doses, though is associated with significant toxicity and has not been effective in improving VEXAS associated cytopenias.

Currently, medical therapeutic options for VEXAS syndrome include IL-1 inhibitors (e.g. Anakinra), IL-6 inhibitors (e.g. Tocilizumab), JAK inhibitors (e.g. Baricitinib), and corticosteroids. Available evidence suggests symptomatic response rates to IL-6 inhibitors and JAK inhibitors are around 50% but that they have no effect on bone marrow failure. Supportive measures include infection prevention through vaccination and prophylactic antibiotics, assessment of bleeding and thrombosis risk, thrombopoetin receptor agonists (e.g. Eltrombopag), erythropoetin, and blood products to optimise blood counts.

For patients with bone marrow failure, HSCT is an option, though case series and case-reported outcomes have been variable. Some patients are reported to have had complete and sustained remission of symptoms and normalisation of cell counts without complication, while others developed significant complications including viral reactivation, graft vs host disease, and in some cases, mortality.

In our case, HSCT was the preferred treatment choice as recurrent infections limited immunosuppression. The patient has been reviewed at day 50 post HSCT. There has been a near resolution of cell counts with Hb 115 g/L, platelets 143 x 10^9/L, WCC 6.1x10^9/L, neutrophils 4.7x10^9/L. Inflammatory symptoms have all but resolved, save persistent paraesthesia in the feet. Tocilizumab has been ceased and prednisolone has been reduced to 4 mg once daily.

**Key learning points:**

• HSCT is an important management option for patients with VEXAS syndrome, however, is associated with significant morbidity and mortality. The nature of VEXAS syndrome means that those affected are more likely to be of more advanced age and frailty, and so at higher risk of post-transplant complications. Some data suggests better outcomes post HSCT in patients who had better controlled disease or less severe disease prior to HSCT and the benefit of early HSCT may be worth considering.

• Further collation of data regarding outcomes of patients who receive HSCT will be beneficial in risk stratifying VEXAS syndrome patients who may be suitable for HSCT.

